# DNA-aware evaluation and debiasing of sequence-to-function models

**DOI:** 10.1093/bioinformatics/btag266

**Published:** 2026-07-07

**Authors:** Doruk Cakmakci, Yue Li

**Affiliations:** School of Computer Science, McGill University, Montreal, QC H3A 0E9, Canada; Mila—Quebec AI Institute, Montréal, QC H2S 3H1, Canada; School of Computer Science, McGill University, Montreal, QC H3A 0E9, Canada; Mila—Quebec AI Institute, Montréal, QC H2S 3H1, Canada

## Abstract

**Motivation:**

Genome sequence-to-function (S2F) models are widely used to interpret base-resolution functional genomics assays. Most S2F models are trained and evaluated against observed counts and profile-shapes using statistical objectives and fidelity metrics. These choices are well motivated, but they are DNA-independent. At the same time, experimental measurements arise from DNA-dependent assays with distinct characteristics. This mismatch motivates a complementary DNA-aware evaluation of S2F-predicted and experimental functional genomic tracks.

**Results:**

We study DNA-dependency of experimental and S2F-predicted tracks using track-conditional genome language models (cgLMs). cgLMs predict masked nucleotides from a conditioning track under controlled DNA visibility. Across ATAC-seq and TF ChIP-seq peaks from GM12878 and K562, cgLM-probing reveals a consistent *masked DNA-decodability gap* between many experimental and S2F-predicted tracks. In particular, single-task (e.g. BPNet) and multi-task (e.g. AlphaGenome) S2F-predicted tracks enabled cgLMs to recover masked nucleotides with significantly higher accuracy and confidence than matched experimental tracks. Analyses of nonpeak and dinucleotide-shuffled sequences show that this gap is not confined to peaks and is not captured by standard DNA-agnostic profile-shape fidelity metrics alone. ChromBPNet Tn5-denoised predictions were an exception and behaved closer to the experimental regime, suggesting that staged training may reduce the gap. We then convert this diagnostic into a critic-derived objective, *DNA-dependency matching* (DDM), using a frozen multi-headed cgLM critic. We introduce Critic-Guided Profile-Shape Editing (CGPSE), a preliminary post hoc debiasing framework for frozen S2F models. In GM12878 ATAC-seq, CGPSE partially reduces the masked DNA-decodability gap for AlphaGenome and BPNet predictions, while exposing a tradeoff with profile-shape fidelity.

**Availability and Implementation:**

https://github.com/li-lab-mcgill/dna-aware-s2f-eval.

## 1 Introduction

Genome sequence-to-function (S2F) prediction models (Avsec *et al.* 2021a, 2021b, Pampari *et al.* 2025, Linder *et al.* 2025, Avsec *et al.* 2026) have become essential for probing DNA sequence determinants of functional genomic assays such as ATAC-seq and transcription factor (TF) ChIP-seq. In essence, these models learn to predict one or more functional genomic tracks at a specified resolution from genomic sequence context. The versatility of these S2F models has enabled downstream applications including variant effect prediction and prioritization (Liu *et al.* 2025), TF motif discovery (Schreiber 2025), and cis-regulatory element design (Schreiber *et al.* 2025).

Existing S2F models typically factorize functional genomics measurements into total observed count and its distribution over the genomic region (i.e. profile-shape). Under this perspective, count and profile-shape predictions are supervised independently (e.g. MSE on log1p total count predictions and multinomial negative log-likelihood on base-resolution profile shape predictions). Similarly, model predictions are evaluated with separate metrics including rank correlation (Spearman/Pearson) for counts and Jensen-Shannon distance (JSD) for profile-shapes. These choices are well motivated and emphasize signal fidelity.

Furthermore, functional genomics measurements arise from DNA-dependent experimental processes (i.e. assays). This dependence is assay-specific. In ATAC-seq, base-resolution profiles reflect Tn5 insertion events along accessible DNA. These events are shaped by local chromatin accessibility and DNA-dependent preferences of Tn5 enzyme. Conversely, in TF ChIP-seq, the DNA-dependency is more indirect. The measurement is driven by TF occupancy, which relies on TF-DNA binding specificity, and the resulting immunoprecipitated enrichment profile. Thus, experimental measurements have distinct relationships to the underlying DNA sequences. However, standard metrics and loss functions used for S2F model training are DNA-independent. This motivates a complementary DNA-aware evaluation of how strongly functional genomic tracks reflect their underlying DNA.

In this work, we study DNA-dependence of experimental and S2F-predicted tracks through the lens of track-conditional genome language models (Nair *et al.* 2025), referred to as cgLMs. Under controlled DNA masking, we quantify DNA-dependence of tracks with cgLM probe-based measurements including masked nucleotide prediction accuracy, cross-entropy, and entropy. We refer to these measurements collectively as *masked DNA-decodability*. Comparing experimental and S2F-predicted tracks across ATAC-seq and TF ChIP-seq data from GM12878 and K562 reveals a consistent *masked DNA-decodability gap* in peak regions. Our initial investigation further shows that this gap is not confined to peak regions and is not captured by standard DNA-agnostic profile-shape fidelity metrics (JSD) alone. Notably, ChromBPNet Tn5-denoised predictions substantially narrow this gap, suggesting that the effect is not inevitable and can be reduced by training design.

We further convert this diagnostic into a critic-derived objective. Specifically, we define *DNA-dependency matching* (DDM), which uses a frozen multi-headed cgLM critic to compare edited S2F predictions with matched experimental tracks under the same DNA-aware probe. Based on this objective, we introduce Critic-Guided Profile-Shape Editing (CGPSE), a preliminary framework for frozen S2F models that edits predicted profile shapes without modifying the underlying S2F model. In GM12878 ATAC-seq, CGPSE partially reduces this gap for AlphaGenome and BPNet predictions, while revealing an important tradeoff with profile-shape fidelity.

## 2 Materials and methods

### 2.1 Datasets

We used publicly available ATAC-seq and TF ChIP-seq measurements for K562 and GM12878 cell lines from ENCODE (ENCODE Project Consortium 2012). The TF ChIP-seq tracks corresponded to CTCF in K562 and ELF1 in GM12878. For each assay, we merged isogenic replicates to obtain consolidated reads. Throughout the experiments, we used chromosomes 8 and 20 for validation, chromosomes 1, 3, and 6 for testing, and the remaining for training.

Merged ATAC-seq BAM files were preprocessed using reads_to_bigwig.py (https://github.com/kundajelab/chrombpnet/blob/master/chrombpnet/helpers/preprocessing/reads_to_bigwig.py) from the ChromBPNet repository with default parameters. This produced a + 4/-4 shift-corrected bigWig file that records base-pair resolution counts at 5’ ends of Tn5 transposase insertions. To ensure standardized comparisons across S2F models, we acquired peak sets and GC-content matched non-peak regions used for training and evaluation of the publicly available ChromBPNet models (K562: ENCFF991RUK; GM12878: ENCFF971WEQ).

For TF ChIP-seq, we generated strand-specific bigWig files that record base-pair resolution counts at 5’ ends of reads. First, we computed bedGraph files from the merged BAM files using bedtools genomecov -5 -bg -strand and converted them to bigWig format with bedGraphToBigWig. For each TF experiment, we obtained the respective IDR thresholded peak set from ENCODE. Using the extract_matching_loci function from the tangermeme package (Schreiber 2025), we sampled non-overlapping nonpeak GC-matched loci to peaks from background regions with low signal relative to peaks.

### 2.2 Sequence-to-function models

For a locus of length *L* base pairs (bp), let $Y\in R_{\geq 0}^{T\times L}$ denote the *T* observed functional genomic tracks, where $Y_{t,\ell }$ is the observed count at position ℓ∈{1,…,L} in track t∈{1,…,T}. Let $X\in {\left\{0,1\right\}}^{4\times L}$ be the one-hot encoded DNA sequence aligned to the locus, and let $X_{f}\in {\left\{0,1\right\}}^{4\times L_{f}}$ be an $L_{f}$-bp flank-extended DNA sequence centered at the locus.

A common S2F modeling choice is to factorize each track $Y_{t}$ as the product of its total count $Y_{t}^{\left(c\right)}=\sum_{\ell =1}^{L}Y_{t,\ell }$ and profile-shape normalized over all positions, $Y_{t,\ell }^{\left(p\right)}=Y_{t,\ell }/Y_{t}^{\left(c\right)}$. With this decomposition, we view an S2F model that predicts *T* tracks as a mapping $f_{s2f}$ from DNA to predicted total counts ${\hat{Y}}^{\left(c\right)}\in R_{\geq 0}^{T}$ and predicted profile-shape distributions ${\hat{Y}}^{\left(p\right)}\in {\left[0,1\right]}^{T\times L}$, and implicitly to predicted tracks $\hat{Y}$:


(1) $$f_{s2f}:X_{f}\mapsto {\hat{Y}}^{\left(c\right)},{\hat{Y}}^{\left(p\right)}\;{\hat{Y}}_{t,\ell }={\hat{Y}}_{t}^{\left(c\right)}\times {\hat{Y}}_{t,\ell }^{\left(p\right)}$$

We consider three S2F model families: BPNet for TF ChIP-seq and ATAC-seq, and ChromBPNet and AlphaGenome for ATAC-seq. All use count/shape factorization and supervise profile-shape predictions with multinomial negative log-likelihood (MNLL), which is equivalent up to an additive constant to a count-weighted cross-entropy loss:


(2) $$L_{\text{CE}}(Y_{t}^{\left(p\right)},{\hat{Y}}_{t}^{\left(p\right)})=-\sum_{\ell =1}^{L}Y_{t,\ell }^{\left(p\right)}\;\text{log}({\hat{Y}}_{t,\ell }^{\left(p\right)})$$

(3) $$\mathrm{MNLL}(Y_{t},{\hat{Y}}_{t}^{\left(p\right)})=Y_{t}^{\left(c\right)}L_{\text{CE}}(Y_{t}^{\left(p\right)},{\hat{Y}}_{t}^{\left(p\right)})$$

This weighting scales the profile-shape loss contribution proportionally with total observed counts, ensuring higher-count loci have a greater impact. The models differ in architecture, number of tracks, and count prediction objective.

#### 2.2.1 TF binding prediction models

We trained BPNet models (Avsec *et al.* 2021b) for TF binding prediction with and without conditioning on control ChIP-seq tracks using bpnet-lite (https://github.com/jmschrei/bpnet-lite). Each model took as input a 2114-bp one-hot DNA sequence and, optionally, a 2114-bp control track, predicted $\text{log}(1+{\hat{Y}}^{\left(c\right)})$, and base-resolution profile shape ${\hat{Y}}^{\left(p\right)}$ for the central 1-kbp region. Both models processed DNA sequences with a backbone of 8 residual dilated convolutional blocks (64 filters per block) to produce a 2,114-by-64 feature map. The models differed in how backbone features were translated into count and profile-shape predictions.

For BPNet without a control-track input, the profile-shape head applied a convolution over backbone features with kernel width of 75, followed by a central crop to the 1-kbp output window. The count head employed a linear projection on backbone features averaged over the same window used by the profile head. For BPNet with control-track input, each prediction head was conditioned separately. For the profile head, stranded control signal was concatenated as additional per-position channels to the backbone feature map before the 75-bp convolution. For the count head, $\text{log}(1+Y_{\text{control}}^{\left(c\right)})$, computed over the count window, was concatenated to the pooled backbone representation before the output projection.

Both models were trained and validated on a 3:1 mixture of peaks and GC-matched nonpeaks, with batch size 64 and learning rate $10^{-3}$, using early stopping (patience 10) for at most 50 epochs. At the start of each epoch, nonpeaks in the training set were resampled with replacement from a larger candidate set to increase background diversity. During training, we used random shift augmentation (±100 bp) and stochastic reverse-complement augmentation (probability 0.5). MSE loss on log1p total count predictions was used as the count fidelity loss, and MNLL was used as the profile-shape fidelity loss.

#### 2.2.2 Chromatin accessibility prediction models

ChromBPNet (Pampari *et al.* 2025) adopts a two-stage, bias-factorized pipeline for modeling ATAC-seq measurements. First, a shallow BPNet-based model $f_{\text{Tn}5}:X_{f}\mapsto {\hat{Y}}_{\text{Tn}5}^{\left(c\right)},{\hat{Y}}_{\text{Tn}5}^{\left(p\right)}$ (4 blocks, 128 filters per block) is trained on a highly inaccessible subset of nonpeak regions (i.e. regions with total count below the 1% quantile of total counts in peak regions). Because of its training data, $f_{\text{Tn}5}$ serves as a proxy for sequence-specific Tn5 enzyme bias in ATAC-seq insertion profiles. In the second stage, a substantially larger BPNet-like model $f_{\text{denoised}}:X_{f}\mapsto {\hat{Y}}_{\text{denoised}}^{\left(c\right)},{\hat{Y}}_{\text{denoised}}^{\left(p\right)}$ (8 blocks, 512 filters per block) is trained together with the frozen Tn5 bias model. Conditioning occurs through output fusion: the total-count predictions from the frozen and trainable models are combined by log-sum-exp, and the profile-shape logits are added before softmax normalization. This staged, bias-factorized training setup shields $f_{\text{denoised}}$ from learning features that explain outputs already captured by $f_{\text{Tn}5}$.

We used both publicly available and in-house trained ChromBPNet models. We downloaded bias-factorized ChromBPNet models for GM12878 (model: ENCSR389HIH) and K562 (model: ENCSR467RSV) from ENCODE. Using a minimally modified version of chrombpnet (https://github.com/kundajelab/chrombpnet), we trained in-house BPNet models architecturally equivalent to $f_{\text{denoised}}$, but without fusion with predictions from the frozen $f_{\text{Tn}5}$ model.

The in-house models were trained and validated on a 10:1 mixture of peaks and GC-matched nonpeaks. Their training setup was matched to the public ChromBPNet configuration and mirrored our BPNet training setup for TF ChIP-seq (Sec. 2.2.1), except for the use of ±500 bp random shift augmentation.

Unlike BPNet and ChromBPNet, which are designed for single-track prediction, AlphaGenome (Avsec *et al.* 2026) is a large genome foundation model trained to predict thousands of tracks across multiple modalities. We queried the AlphaGenome API via predict_interval to obtain ATAC-seq predictions for a 2,048-bp region centered on each peak and nonpeak sequence for GM12878 (EFO : 0002784) and K562 (EFO : 0002067).

### 2.3 Track conditional genome language models

For a locus of length *L* bp, let $X\in {\left\{0,1\right\}}^{4\times L}$ and $\tilde{Y}\in R_{\geq 0}^{T\times L}$ denote the DNA sequence and *T* functional genomic tracks (e.g. experimental *Y* or S2F-predicted $\hat{Y}$), respectively. Conceptually, cgLMs correspond to the functional language modeling mode of Nona (Nair *et al.* 2025), a multimodal masked genome modelling framework.

cgLMs can be viewed as a mapping $f_{\mathrm{cgLM}}$ from conditioning tracks $\tilde{Y}$ and masked DNA $f_{\text{mask}}(X,M_{i})$ to predicted nucleotide probabilities for all input positions $\hat{X}\in {\left[0,1\right]}^{4\times L}$:


(4) $$f_{\mathrm{cgLM}}:\tilde{Y},f_{\text{mask}}(X,M_{i})\mapsto \hat{X}$$

Here, $M_{i}\in {\left\{0,1\right\}}^{L}$ is the DNA input mask and $f_{\text{mask}}$ is the masking function of choice. $M_{i}[\ell ]=1$ indicates that the nucleotide at position ℓ is masked from cgLM input.

Similar to their DNA-only counterparts, cgLMs are trained with a masked cross-entropy objective between predicted nucleotide probabilities $\hat{X}$ and the reference genome *X*:


(5) $$L_{\text{MLM}}=-\frac{\sum_{\ell =1}^{L}M_{o}[\ell ]\sum_{b=1}^{4}X_{b,\ell }\;\text{log}\;{\hat{X}}_{b,\ell }}{\sum_{\ell =1}^{L}M_{o}[\ell ]}$$

Here, $M_{o}\in {\left\{0,1\right\}}^{L}$ is the supervision mask, where $M_{o}[\ell ]=1$ indicates that position ℓ contributes to the loss. In practice, the relationship between $M_{o}$ and $M_{i}$ can follow either tied (i.e. $M_{o}\equiv M_{i}$) or nested (i.e. $M_{o}\subseteq M_{i}$) masking schemes.

In this work, we considered two cgLM model families: single-headed cgLMs and multi-headed cgLM critics. These models were trained on experimental *Y* and S2F-predicted $\hat{Y}$ tracks for ATAC-seq (T=1, unstranded) and TF ChIP-seq (T=2, stranded). Single-headed cgLMs were trained on one conditioning-track source at a time (i.e. *Y* or $\hat{Y}$), whereas multi-headed cgLM critics were trained jointly on both sources. Unless otherwise specified, all models were trained on 1-Kbp loci with tied input and supervision masks ($M_{o}\equiv M_{i}$).

For both model families, each locus in a training batch was assigned a DNA masking rate $r∼U(r_{\min},r_{\max})$, and n=⌊rL⌋ nucleotide positions were masked uniformly without replacement. We implemented $f_{\text{mask}}$ by multiplicative masking with $\left(1-M_{i}\right)$ followed by concatenation of $M_{i}$ as an explicit mask-indicator channel, yielding a masked DNA tensor of shape 2×L×4. Conditioning tracks remained fully visible to the models, but were passed through the same transformation with a zero mask, yielding tensors of shape 2×L×T. During training, $L_{\text{MLM}}$ was first computed per sequence and then averaged across the batch.

For ATAC-seq, cgLMs were trained on a 10:1 mixture of peaks and GC-matched nonpeaks with shift augmentation of ±500 bp. For TF ChIP-seq, cgLMs were trained on a 3:1 mixture of peaks and GC-matched nonpeaks with shift augmentation of ±100 bp. Training-set nonpeak regions were resampled at the beginning of each epoch to increase background diversity. Validation-set nonpeak regions were subsampled once before model training.

#### 2.3.1 Single-headed cgLM models

**Figure 1:**
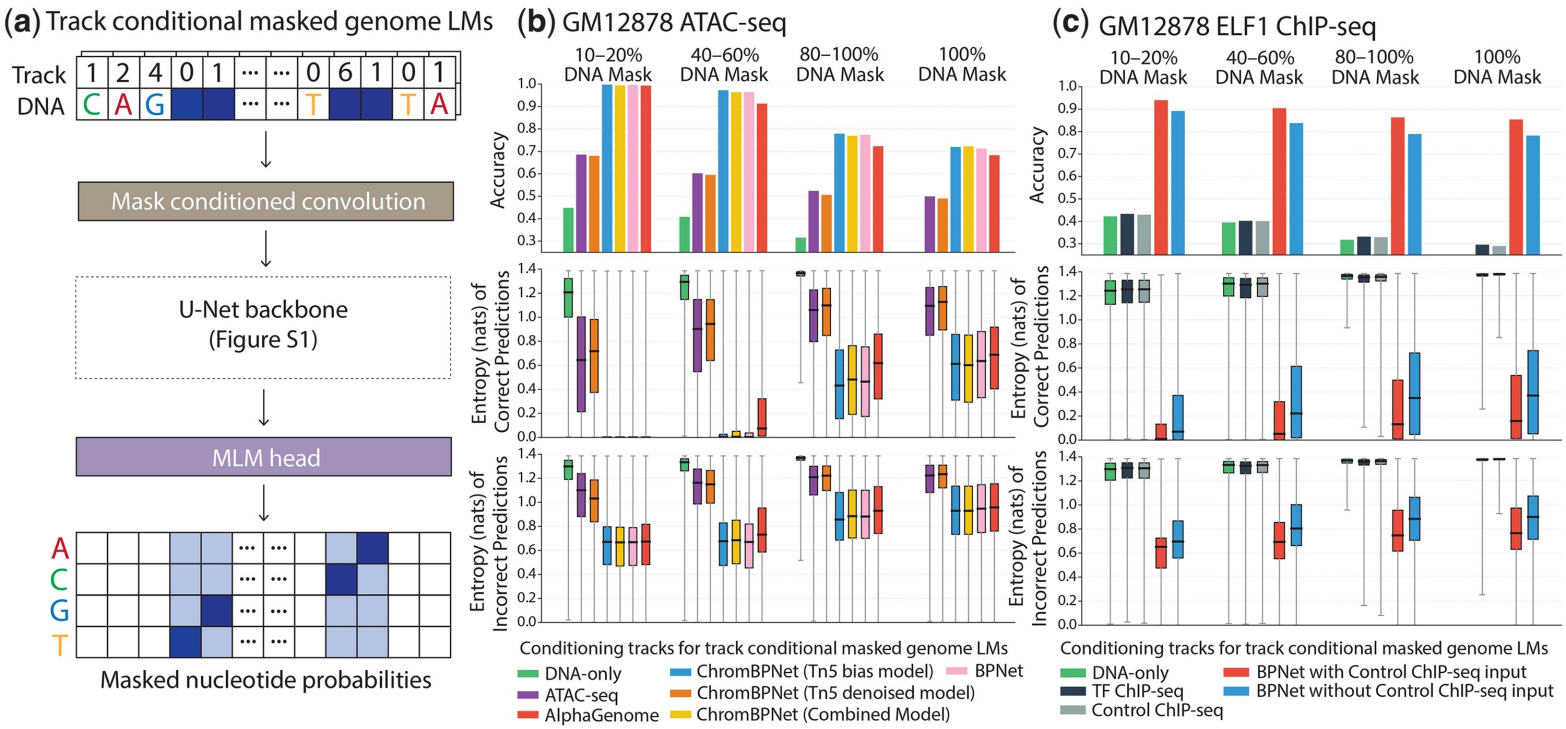
cgLMs reveal a masked DNA-decodability gap between experimental and S2F-predicted tracks in held-out peak regions. (a) cgLMs map tracks and masked DNA to per-position probabilities over the four nucleotides. (b) Test set (peaks) performance of cgLM and DNA-only baseline (green) trained on GM12878 ATAC-seq (purple) as well as S2F-predicted tracks (others) across DNA masking regimes. Each bar corresponds to a cgLM model under stated masking regime and colors denote the conditioning tracks. (c) Test set (peaks) performance of cgLM and DNA-only baselines trained on GM12878 ELF1 ChIP-seq (black), control track (grey) and predictions from BPNet with (red) and without (blue) control input. Each bar corresponds to a cgLM model under stated masking regime and colors denote the conditioning tracks.

We adopted a U-Net-based architecture with 2.6M parameters for single-headed cgLM models (Fig. 1a). The model operates on concatenated masked DNA and conditioning tracks of shape B×2×L×(4+T), and predicts nucleotide probabilities of shape B×L×4. The model employs three down blocks to reduce input length by a factor of 8, two bottleneck blocks with transformers for global mixing, and three up blocks with skip connections to upsample the mixed representations to the input length, followed by an MLM head for prediction. For DNA-only baselines, conditioning tracks were masked in the input using an all-ones mask. The complete architectural specification is provided in Fig. S1, available as supplementary data at *Bioinformatics* online.

Single-headed cgLMs were trained under four DNA masking-rate regimes with tied DNA input and supervision masks, $M_{o}^{\left(X\right)}\equiv M_{i}^{\left(X\right)}$: 10%–20%, 40%–60%, 80%–100%, and 100% masked DNA (track-only). All single-headed cgLMs were trained with AdamW (learning rate $10^{-3}$, weight decay $10^{-3}$) for up to 200 epochs with early stopping (patience 10), while monitoring masked cross-entropy on held-out peak regions.

#### 2.3.2 Multi-headed cgLM critics

We adopted a U-Net-based architecture with 2.7M parameters for multi-headed cgLM critics (Fig. 3a). The critic backbone processes masked DNA of shape 2×L×4 and tracks of shape 2×L×T with separate stems, followed by additive fusion, down blocks, bottleneck blocks, and up blocks. The resulting backbone embeddings of shape B×L×D are provided to three heads: an experimental-track MLM head, an S2F-track MLM head, and an embedding source classifier (experimental vs S2F). The complete architectural specification is provided in Fig. S2, available as supplementary data at *Bioinformatics* online.

The multi-headed cgLM critic training setup mirrored that of single-headed cgLMs with a few exceptions. During training, the critic operated on experimental and S2F-predicted tracks independently, and backbone embeddings were routed to the respective MLM heads. To isolate differences between S2F and experimental tracks in their profile shapes, S2F-track total counts were rescaled to match those of the corresponding experimental tracks. The critic objective was the equally weighted sum of the experimental-head MLM loss, the S2F-head MLM loss, and the discriminator loss, each computed over masked positions and averaged across the batch. The critic was trained with a wider DNA masking-rate range (10%–100%) for 300 epochs without early stopping, and validation performance was monitored across six mask bins for each head.

### 2.4 Critic-Guided Profile Shape Editing

CGPSE is a post hoc framework for editing profile-shape predictions from frozen S2F models to reduce the masked DNA-decodability gap. It uses a frozen multi-headed cgLM critic to define the DNA-dependency matching (DDM) objective. The trainable editor has two stages, a DNA-free stage and a DNA-aware stage, and is summarized in Fig. 4.

#### 2.4.1 Profile-shape representation and editor mapping

Using the count/profile-shape factorization introduced in Sec. 2.2, let $Y^{\left(p\right)}$ and ${\hat{Y}}^{\left(p\right)}$ denote the experimental and frozen S2F-predicted profile-shape distributions, respectively. We define the corresponding log profile shapes as $S=\text{log}(Y^{\left(p\right)}+\varepsilon )$ and $\hat{S}=\text{log}({\hat{Y}}^{\left(p\right)}+\varepsilon )$. The DNA-free editor maps the S2F-predicted log profile shape to an edited log profile shape together with a latent representation. The DNA-aware editor refines the DNA-free edited output using the latent representation and masked DNA. Both stages can be formulated as:


(6) $$f_{\text{free}}:\hat{S}\mapsto {\tilde{S}}_{\text{free}},K_{\text{free}}$$

(7) $$f_{\text{aware}}:{\tilde{S}}_{\text{free}},K_{\text{free}},f_{\text{mask}}(X,M_{\text{edit}})\mapsto {\tilde{S}}_{\text{aware}},K_{\text{aware}}$$

(8) $${\tilde{Y}}_{\text{free}}^{\left(p\right)}=\text{softmax}({\tilde{S}}_{\text{free}})\;{\tilde{Y}}_{\text{aware}}^{\left(p\right)}=\text{softmax}({\tilde{S}}_{\text{aware}})$$

Here, $K_{\text{free}}\in R^{C\times N}$ and $K_{\text{aware}}\in R^{C\times N}$ denotes the latent representation produced by the DNA-free and DNA-aware editors, respectively. $M_{\text{edit}}\in {\left\{0,1\right\}}^{L}$ denotes the DNA input mask used by the DNA-aware editor.

#### 2.4.2 DNA-dependency matching objective

Let $C^{\;\text{exp}\;}$ denote the frozen experimental-track head of the multi-headed cgLM critic. Consistent with Sec. 2.3, $C^{\;\text{exp}\;}$ maps a conditioning track and masked DNA to nucleotide probabilities $\hat{X}\in {\left[0,1\right]}^{4\times L}$. Applying $C^{\;\text{exp}\;}$ to the experimental track *Y* and an edited track $\tilde{Y}$ yields critic outputs ${\hat{X}}^{\;\text{exp}\;}$ and ${\hat{X}}^{\text{edit}}$, respectively.

DDM is computed at positions indicated by the critic supervision mask $M_{o}$. The loss has three terms. The KL term aligns full nucleotide distributions. The entropy-matching term aligns confidence when the most probable nucleotide identities agree. The reference term penalizes excessive reference-nucleotide confidence when the experimental-track critic output is maximized at the reference nucleotide. For position ℓ, let $b_{\ell }^{\;\text{exp}\;}=\arg\max_{b}{\hat{X}}_{\ell }^{\;\text{exp}\;}(b)$, $b_{\ell }^{\text{edit}}=\arg\max_{b}{\hat{X}}_{\ell }^{\text{edit}}(b)$, and $b_{\ell }^{\text{ref}}=\arg\max_{b}X_{b,\ell }$. We denote Shannon entropy by H(·) and the Huber loss with threshold δ by $h_{\delta }(\cdot )$.


(9) $$L_{\text{KL}}=\frac{1}{\left|M_{o}\right|}\sum_{\ell \in M_{o}}\text{KL}\left({\hat{X}}_{\ell }^{\;\text{exp}\;}\;||\;{\hat{X}}_{\ell }^{\text{edit}}\right)$$

(10) $$L_{H}=\frac{1}{\left|M_{o}\right|}\sum_{\ell \in M_{o}}1\left[b_{\ell }^{\;\text{exp}\;}=b_{\ell }^{\text{edit}}\right]{\left(H({\hat{X}}_{\ell }^{\;\text{exp}\;})-H({\hat{X}}_{\ell }^{\text{edit}})\right)}^{2}$$

(11) $$\Delta_{\ell }^{\text{ref}}=-\text{log}\;{\hat{X}}_{\ell }^{\text{edit}}(b_{\ell }^{\text{ref}})+\text{log}\;{\hat{X}}_{\ell }^{\;\text{exp}\;}(b_{\ell }^{\text{ref}})$$

(12) $$L_{\text{REF}}=\frac{1}{\left|M_{o}\right|}\sum_{\ell \in M_{o}}1\left[b_{\ell }^{\;\text{exp}\;}=b_{\ell }^{\text{ref}}\right]h_{\delta }(\text{ReLU}(\Delta_{\ell }^{\text{ref}}))$$

(13) $$L_{\text{DDM}}=L_{\text{KL}}+L_{H}+L_{\text{REF}}$$

During CGPSE, gradients flow only into the edited track branch, and the critic is never updated. Exact implementation details are provided in Supplementary Text Sec. S1.1, available as supplementary data at *Bioinformatics* online.

#### 2.4.3 DNA-free profile-shape editor training

The DNA-free profile-shape editor is a debiasing autoencoder (DAE) that operates only on log profile-shapes. It maps the S2F-predicted log profile shape $\hat{S}$ to an edited log profile shape ${\tilde{S}}_{\text{free}}$ together with a latent representation $K_{\text{free}}$. Experimental profile-shape inputs are reconstructed, whereas S2F-predicted profile-shape inputs are mapped to the matching experimental profile shapes; the two routes share a decoder but use separate encoders. The latent representations produced by the encoders are aligned to encourage a shared latent space between experimental and S2F profile-shapes. DDM supervises outputs from both modes under the frozen critic.

#### 2.4.4 DNA-aware profile-shape editor training

The DNA-aware profile-shape editor builds on the trained DNA-free DAE and introduces DNA as a correction signal in its latent space. In this stage, the DNA-free DAE is frozen. The trainable components are a profile-shape re-encoder, a masked-DNA encoder, and a latent editor that employs Feature-wise Linear Modulation (Perez et al. 2018) . The re-encoder and masked-DNA encoder jointly produce a context representation, which the latent editor combines with $K_{\text{free}}$ to update the latent representation before decoding.

To limit direct copying of visible DNA into edited outputs, editor DNA visibility is coupled to critic supervision through $M_{\text{edit}}=\neg M_{o}$. As a result, positions whose DNA is visible to the editor are simultaneously supervised by DDM through the frozen critic.

#### 2.4.5 Shared training objective

As discussed in Sec. 2.2, MNLL is equivalent to multiclass cross entropy with linear total-count weighting (Equation 3). CGPSE uses the same cross-entropy form, but replaces linear weighting with a sublinear count coefficient $w(Y^{\left(c\right)})=\sqrt{\max(Y^{\left(c\right)},c_{\min})}$ where $c_{\min}$ is the 75th percentile of total counts across matched nonpeak loci. This keeps high-count peaks upweighted, but reduces their dominance during optimization. For both editor variants, the full training objective is


(14) $$L_{\text{total}}=w(Y^{\left(c\right)})(L_{\text{CE}}+L_{\text{latent}}+\lambda_{t}L_{\text{DDM}})$$

Here, $L_{\text{CE}}$ denotes a multiclass cross-entropy loss on profile shapes (Equation 2), $L_{\text{latent}}$ denotes a latent alignment loss, and $L_{\text{DDM}}$ denotes the DNA-dependency matching objective (Equation 13). The coefficient $\lambda_{t}$ is adapted online with a gradient-ratio controller.

The two editor variants differ in loss routing. In the DNA-free stage, reconstruction and DDM losses are applied to both experimental and S2F-input modes, while $L_{\text{latent}}$ aligns their latent representations. In the DNA-aware stage, the experimental route provides a fixed reference, and all three terms are applied only to the edited S2F branch. Additional implementation details are provided in Supplementary Text Sec. S1.1, available as supplementary data at Bioinformatics online.

## 3 Results

### 3.1 Experimental track conditional genome language modeling

We used single-headed cgLM models (Fig. 1a, Sec. 2.3.1) to quantify how well masked nucleotides can be recovered from base-resolution ATAC-seq and TF ChIP-seq measurements in GM12878 and K562 (Sec. 2.1). For each experiment, we trained separate models under four DNA-masking regimes (10%–20%, 40%–60%, 80%–100%, and 100% DNA mask, Sec. 2.3.1). Model performance was assessed on 1-Kbp peak-centered regions in held-out chromosomes with masked nucleotide prediction accuracy. Model confidence was summarized by the entropy (nats) of the predicted nucleotide distribution at masked positions. Entropy was reported separately for correctly and incorrectly predicted sites.

For GM12878 ATAC-seq peaks (Fig. 1b), a track-only cgLM (100% DNA mask) can recover masked nucleotides from Tn5 transposase insertion profiles with ∼50% accuracy, well above chance (∼25%). Comparatively, a DNA-only MLM baseline with light DNA masking (10%–20%) is capped at ∼45% accuracy, and is less confident in its correct predictions (higher entropy). Thus, cgLM models recognize substantial DNA-informative patterns in Tn5 insertion profiles, which provide decisive evidence for some masked bases even without sequence input.

cgLM performance improves further when DNA and Tn5 insertion profiles are provided together (Fig. 1b). Masked nucleotide recovery from Tn5 insertion profiles becomes easier with increasing DNA visibility, up to ∼68% accuracy at 10–20% DNA masking. cgLM confidence in correct predictions follows a similar trend: The median entropy decreases from ∼1.09 to ∼0.67 nats. Therefore, cgLM models recognize complementary DNA-informative patterns from Tn5 insertion profiles and DNA sequences.

In contrast to ATAC-seq, employing cgLMs on the ELF1 TF ChIP-seq and its control ChIP-seq tracks shows much weaker masked nucleotide recovery (Fig. 1c). Across DNA masking rates, performance and confidence of cgLMs on ChIP-seq tracks are similar to DNA-only MLM baseline. Moreover, performance of track-only cgLMs remains close to chance (25% accuracy) with near-maximal entropy (∼1.386 nats). This modality dependence of cgLM performance and confidence is consistent with known differences between assays. ATAC-seq provides dense, base-resolved accessibility structure across peak regions. TF and control ChIP-seq profiles are comparatively sparse and motif-centered, and therefore constrain nucleotide identity much more weakly at base resolution.

These assay-dependent findings reproduce on peaks from held-out chromosomes across cell lines. GM12878 and K562 ATAC-seq mirror each other in accuracy and entropy profiles (Fig. 1b; Fig. S4a, available as supplementary data at *Bioinformatics* online). Moreover, GM12878 ELF1 and K562 CTCF ChIP-seq settings exhibit similar behavior (Fig. 1c; Fig. S4b, available as supplementary data at *Bioinformatics* online). Together, our findings position cgLM as a stable and assay-consistent DNA-aware probe of DNA–track dependence.

### 3.2 Masked DNA-decodability gap between experimental and S2F-predicted tracks

Using the single-headed cgLM probe introduced in Sec. 3.1, we compared DNA–track dependence between S2F-predicted and experimental tracks. For this comparison, we trained a separate set of cgLMs conditioned on S2F-predicted tracks. We quantified the differences in DNA–track dependence using masked nucleotide prediction accuracy and prediction confidence under the cgLM probe. This analysis provides a DNA-aware comparison of S2F predictions and experimental tracks that complements track-only metrics such as JSD.

For GM12878 ATAC-seq peaks (Fig. 1b), cgLMs trained on AlphaGenome and BPNet predictions achieve substantially higher masked nucleotide prediction accuracy than cgLMs trained on experimental tracks. This separation is present across all DNA visibility settings. In the track-only regime (100% DNA mask), predicted-track cgLMs reach ∼68%–72% accuracy, compared with ∼50% for the experimental-track cgLM. Under partial DNA masking, the gap widens further. Notably, under light DNA masking (10%–20% DNA mask), cgLMs with S2F-predicted conditioning tracks achieve near-perfect masked DNA recovery (≳99%). cgLM prediction confidence follows this trend. Under light DNA masking, entropy of predictions from S2F-predicted conditioning tracks collapses toward zero (median $\approx 10^{-2}$ nats). However, entropy of predictions from experimental conditioning tracks retains a broader distribution.

We observe a similar pattern for GM12878 ELF1 ChIP-seq (Fig. 1c). cgLMs trained on BPNet-predicted tracks achieve substantially higher masked nucleotide prediction accuracy than cgLMs trained on experimental ELF1 or control ChIP-seq tracks (e.g. ∼90% versus ∼42% at 10%–20% DNA mask; Sec. 3.1). Accordingly, the difference in cgLM prediction confidence on S2F-predicted and experimental conditioning tracks mirror prediction accuracy.

We refer to this systematic separation in cgLM performance and confidence as a *masked DNA-decodability gap* between experimental and S2F-predicted tracks. The gap is already present in the track-only regime (100% DNA mask) and becomes extreme once modest DNA context is revealed to cgLMs (40%–60% and 10%–20% DNA mask). Thus, under the cgLM probe, S2F-predicted tracks support substantially easier masked nucleotide recovery than matched experimental tracks.

We observe consistent behavior in an independent setting (K562 ATAC-seq and CTCF ChIP-seq), supporting the reproducibility of these findings across cell lines and assay types (Fig. S4, available as supplementary data at *Bioinformatics* online). Together, these results establish a robust masked DNA-decodability gap between experimental and S2F-predicted tracks that persists across DNA visibility regimes, assay types, and biological context.

### 3.3 Initial investigation of the masked DNA-decodability gap

To better understand the masked DNA-decodability gap between experimental and S2F-predicted tracks (Sec. 3.2), we performed an initial investigation that constrains how the gap should be interpreted.

We first examined cgLMs trained on predictions from bias-factorized ChromBPNet and its sub-models (Sec. 2.2.2). Across GM12878 and K562 ATAC-seq, cgLMs trained on predictions from the *nonpeak-trained* ChromBPNet Tn5 bias model remained highly decodable on peaks, comparable to BPNet and AlphaGenome (Fig. 1b; Fig. S4a, available as supplementary data at *Bioinformatics* online). This suggests that the highly decodable DNA-linked structure captured by the bias branch transfers from the nonpeak regime used for S2F training to peak regions used for cgLM evaluation. In contrast, cgLMs trained on ChromBPNet Tn5-denoised predictions showed substantially reduced masked DNA decodability and were closer to the experimental ATAC regime in both cell lines (Fig. 1b; Fig. S4a, available as supplementary data at *Bioinformatics* online), suggesting that the masked DNA-decodability gap can be substantially reduced by a staged training formulation.

**Figure 2:**
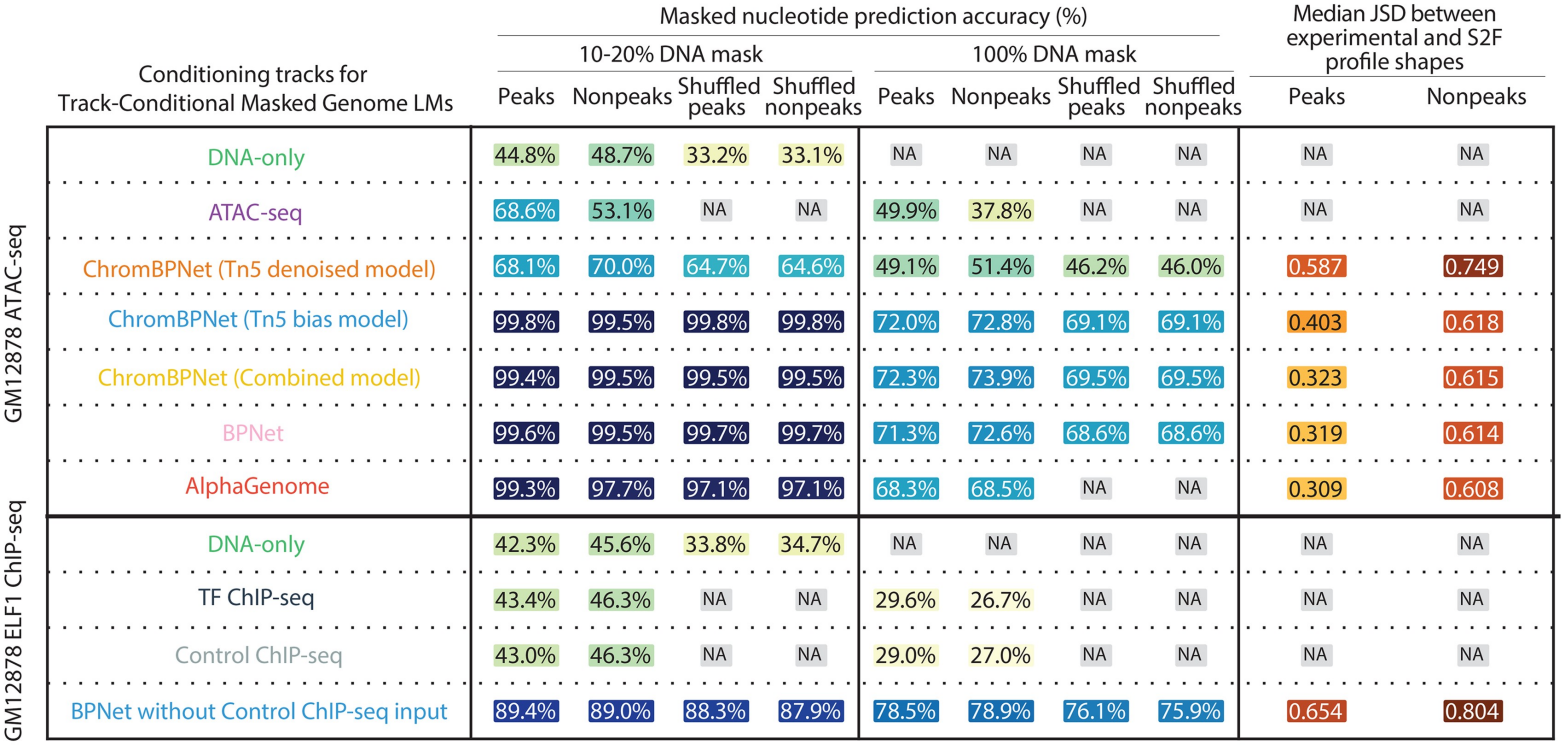
cgLMs recover GM12878 peak, nonpeak and shuffled DNA sequences from S2F-predicted tracks with comparable accuracy. Rows are grouped by assay (GM12878 ATAC-seq and ELF1 ChIP-seq), and list the conditioning tracks used for track-conditional genome language models. The upper block shows GM12878 ATAC-seq. The lower block shows GM12878 ELF1 ChIP-seq. Columns are grouped into masked nucleotide prediction accuracy at 10%–20% DNA mask and at 100% DNA mask, each with separate subcolumns for peaks, nonpeaks, shuffled peaks, and shuffled nonpeaks. The final column group reports the S2F predicted profile-shape fidelity metric (median JSD) on peaks and nonpeaks. NA indicates settings not applicable (e.g. shuffled sequence evaluation with experimental assay tracks).

We then re-evaluated trained cgLMs on held-out peaks, nonpeaks, and their dinucleotide-preserving shuffles in both cell lines (Fig. 2; Fig. S5, available as supplementary data at *Bioinformatics* online), focusing on light DNA masking (10%–20%) and the track-only regime. In both cell lines, experimental ATAC-seq tracks were more decodable on peaks than on nonpeaks, whereas experimental TF ChIP-seq and control ChIP-seq tracks remained weak overall, especially in the track-only setting. The DNA-only baseline also dropped on shuffled loci, confirming that shuffling disrupted recoverable sequence structure. However, many cgLMs trained on S2F-predicted tracks remained highly decodable across peaks, nonpeaks, and shuffled loci, with much weaker regime dependence than the matched experimental tracks. ChromBPNet Tn5-denoised was a meaningful partial exception: it reduced masked DNA decodability toward the experimental ATAC regime, but still showed relatively similar behavior across peaks, nonpeaks, and shuffled loci. Overall, these results show that the effect is not restricted to peaks and cannot be fully explained by assay-specific differences. Instead, many S2F-predicted profile shapes appear to retain substantial input-DNA information under the cgLM probe, even at nonpeak and shuffled loci.

We next compared masked DNA decodability with profile-shape fidelity measured by JSD. Across both GM12878 and K562 ATAC-seq, the median nonpeak JSD was worse than the peak JSD for all S2F models. Yet, masked DNA decodability remained nearly unchanged across the same regions. ChromBPNet Tn5-denoised made this distinction especially clear: among the ATAC S2F models, it showed the lowest masked DNA decodability while also having the highest JSD. Thus, masked DNA decodability captures a DNA-aware property of predicted tracks that is not captured by DNA-agnostic fidelity metrics such as JSD.

Finally, we performed three targeted control experiments to ablate certain design decisions of our cgLM training setup (Supplementary Text S1.2, available as supplementary data at *Bioinformatics* online). Together, we observed that (1) discrepancy between S2F-predicted and experimental profile-shapes is the main driver of masked DNA-decodability gap (Fig. S6a, available as supplementary data at *Bioinformatics* online); (2) cgLM training on coarser resolutions (down to 8-bp) suggests the sequence-linked structure is observable at multiple-resolutions but concentrated at finer scales (Fig. S6b, available as supplementary data at *Bioinformatics* online); and (3) Screening a range of window lengths (16 to 512-bp) with cgLMs indicate that the structure is auditable to a substantial extent in 16-bp windows but benefits from longer range context (Fig. S6c, available as supplementary data at *Bioinformatics* online).

### 3.4 Multi-headed cgLM critic captures the masked DNA-decodability gap

We next sought an instrument that could jointly process experimental and S2F-predicted tracks within a single model, and capture the masked DNA-decodability gap across DNA-visibility regimes.

**Figure 3:**
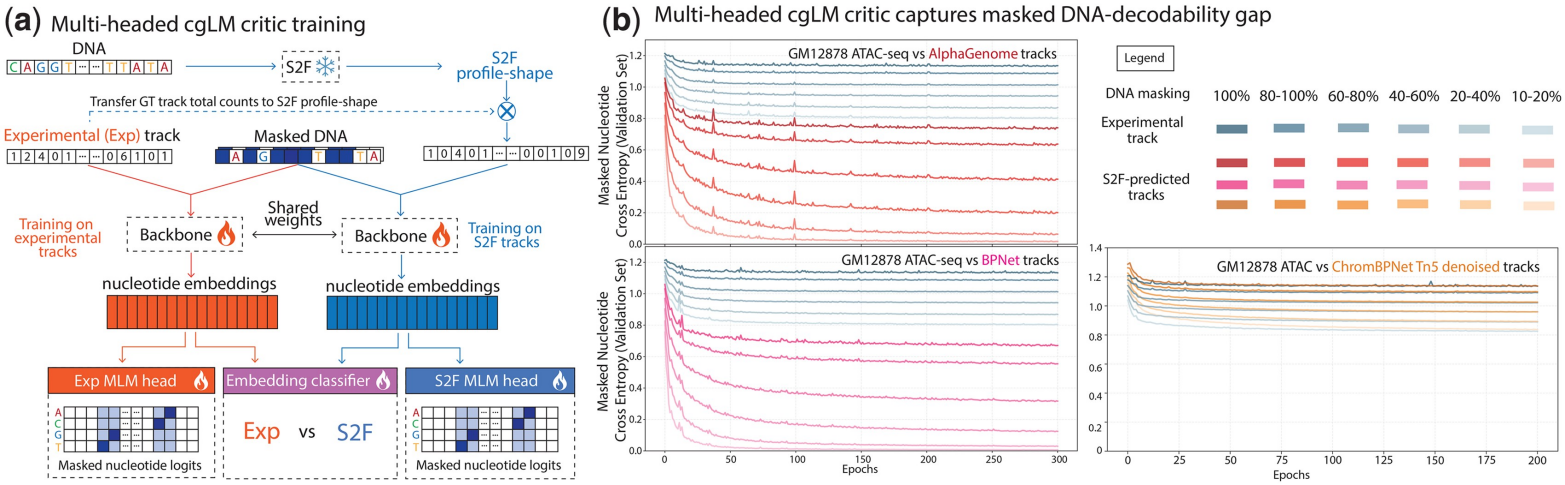
Multi-headed cgLM critic captures masked DNA-decodability gap (a) Multi-headed cgLM processes experimental (Exp) and total count matched S2F inputs with a shared source-agnostic backbone. During training, the backbone embeddings are routed to source-specific MLM heads, and a source classifier. (b) Multi-headed cgLM critic performance for GM12878 ATAC-seq vs AlphaGenome (top left), BPNet (bottom left) and ChromBPNet Tn5-denoised (bottom right) on validation peaks. The masked DNA-decodability gap is quantified as the difference in cross entropy loss between experimental and S2F sources.

For this purpose, we designed a multi-headed cgLM critic trained jointly on S2F-predicted and experimental (Exp) tracks (Fig. 3a; Sec. 2.3.2), with a backbone that does not distinguish between S2F and experimental track inputs. During training, backbone outputs are routed to source-specific MLM heads and a source discriminator, allowing source specialization. In addition, S2F-predicted inputs were rescaled to match the total counts of the corresponding experimental tracks. With this design, divergence between the experimental and S2F MLM heads is attributable primarily to systematic profile-shape differences rather than to total-count differences.

We trained multi-headed cgLM critics on GM12878 ATAC-seq versus AlphaGenome, BPNet, and ChromBPNet Tn5-denoised predictions under 10%–100% DNA masking regime. In this setting, we quantified the masked DNA-decodability gap across DNA-visibility regimes as the difference between the masked mean cross-entropy of the Exp and S2F MLM heads on validation peaks (Fig. 3b). For AlphaGenome and BPNet versus GM12878 ATAC-seq, the critic recapitulated the masked DNA-decodability gap. In contrast, the critic for ChromBPNet Tn5-denoised versus GM12878 ATAC-seq showed minimal drift between heads. These trends are coherent with Sec. 3.2 and show that a single multi-headed critic can capture the masked DNA-decodability gap across sources and DNA-visibility regimes.

### 3.5 Critic-Guided Profile-Shape editing

**Figure 4:**
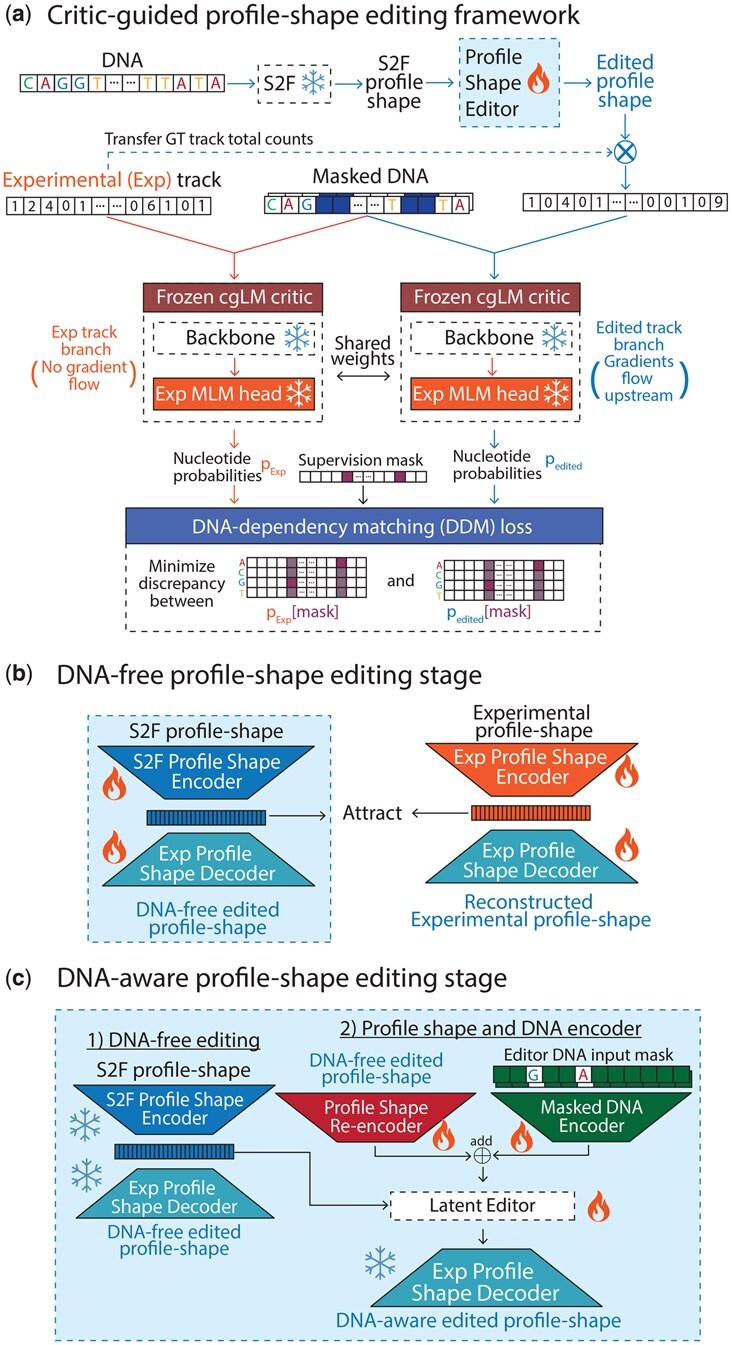
CGPSE overview. (a) Critic-guided framework. A frozen S2F model produces a profile-shape prediction, which is edited and combined with matched experimental total counts. The frozen multi-headed cgLM critic is then applied to the edited track and the matching experimental track under masked DNA. DNA-dependency matching (DDM) minimizes discrepancies between nucleotide probabilities predicted by the experimental (Exp) MLM head on the two inputs, and gradients flow only through the edited-track branch. (b) DNA-free profile-shape editing stage. A DNA-free debiasing autoencoder (DAE) is trained in two modes: experimental profile-shapes are reconstructed, whereas S2F-predicted profile-shapes are mapped to matched experimental profile-shapes. The two routes share a decoder and are trained jointly with DDM, profile-shape fidelity, and a latent alignment loss that encourages a shared latent space. (c) DNA-aware profile-shape editing stage. The frozen DNA-free DAE output is re-encoded together with masked DNA to produce a correction signal. A latent editor combines this signal with the frozen DNA-free latent representation and updates it before decoding, yielding a DNA-aware edited profile-shape. The learnable modules are trained with the same objectives as DNA-free DAE.

The ability of multi-headed cgLM critics to measure the masked DNA-decodability gap (Sec. 3.4) suggests that the same signal may be used to reduce it. We therefore developed CGPSE (Fig. 4), a preliminary framework for editing profile-shape predictions from frozen, black-box S2F models. All CGPSE experiments were performed on AlphaGenome and BPNet predictions for GM12878 ATAC-seq (T=1, L=1000).

We first asked whether the masked DNA-decodability gap could be reduced with a profile-shape editor that does not receive DNA explicitly. To test this, we used a DNA-free profile-shape editor (Fig. 4b) implemented as a debiasing autoencoder (DAE; Sec. 2.4.3; Fig. S3a, available as supplementary data at *Bioinformatics* online) and trained with a weighted mix of profile-shape fidelity and DDM losses (Sec. 2.4.5). Following Sec. 2.4.5, we retained the same cross-entropy form as MNLL (Equation 3), but replaced linear count weighting with a sublinear coefficient equal to the square root of total count. This change was motivated by the comparable prominence of the masked DNA-decodability gap in peak and nonpeak regions (Sec. 3.3) and by the need to reduce the dominance of the highest-count loci during joint optimization with DDM.

**Figure 5:**
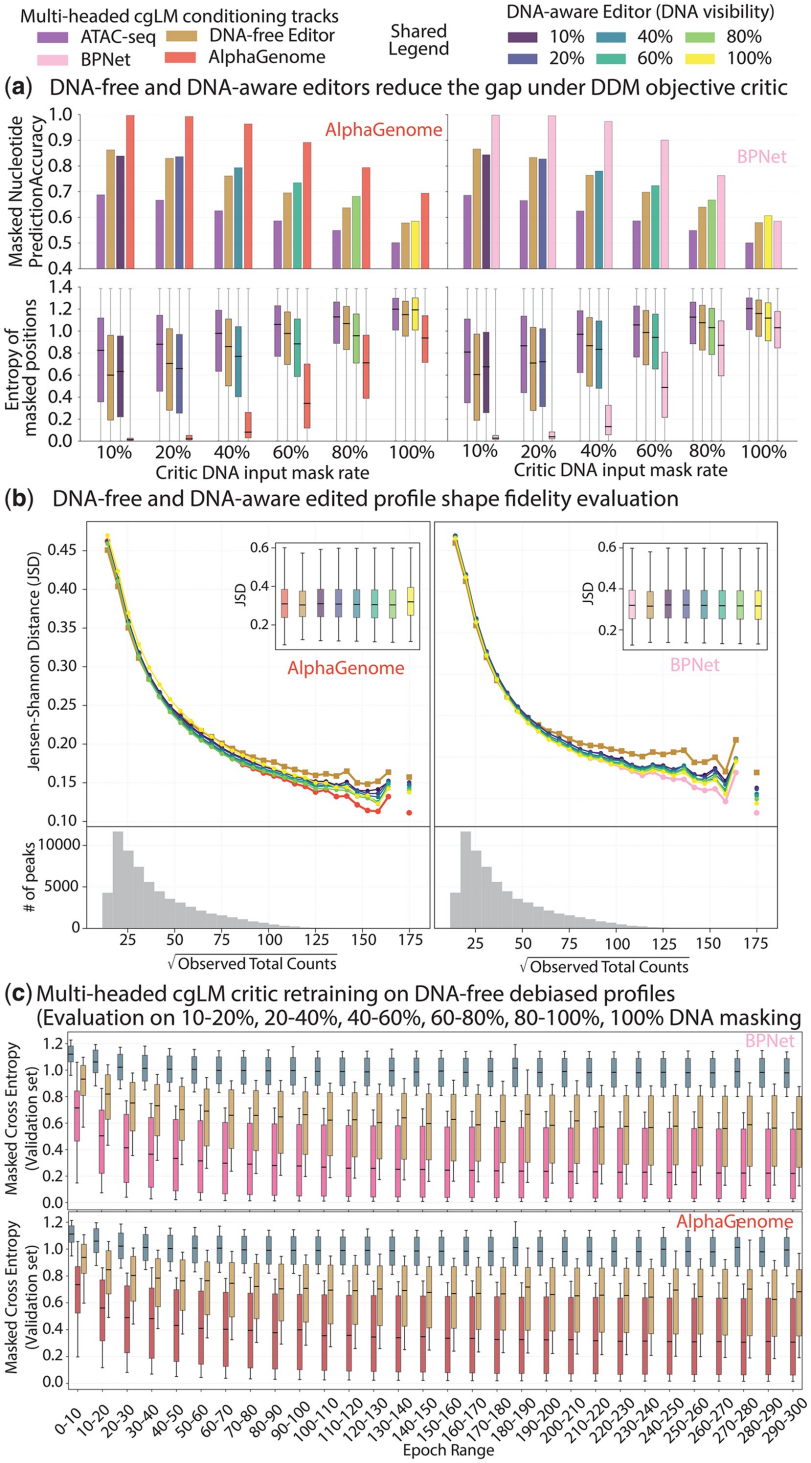
CGPSE application on AlphaGenome and BPNet predictions for GM12878 ATAC-seq (a) DNA-free and DNA-aware editors reduce but do not remove the masked DNA-decodability gap under the critic used for training. For AlphaGenome (red) and BPNet (pink) predictions, masked nucleotide prediction accuracy (top) and entropy at masked positions (bottom) are shown across critic DNA input mask rates for the experimental ATAC-seq track (purple), the original S2F prediction, and the corresponding edited tracks. (b) Profile-shape fidelity of edited tracks, measured by JSD and stratified by the observed square root of total count. Fidelity degradation is concentrated in stronger peaks, whereas lower-count regions are affected less. DNA-aware editing partially recovers fidelity relative to the DNA-free stage. Insets summarize the JSD distributions without stratification. (c) Retraining a multi-headed cgLM critic on DNA-free edited profiles as a robustness control. Boxes summarize masked cross-entropy computed on validation set across non-overlapping 10-epoch windows for the 10%–20%, 20%–40%, 40%–60%, 60%–80%, 80%–100%, and 100% DNA masking regimes. Performance curves before windowing are provided in Fig. S7, available as supplementary data at *Bioinformatics* online.

Under the critic used for DDM, DNA-free editing reduced the masked DNA-decodability gap for both S2F models (Fig. 5a). However, this reduction came with a fidelity cost, most notably in stronger peaks when profile-shape fidelity was stratified by the observed square root of total count (Fig. 5b). This tradeoff is consistent with the change from linear to sublinear weighting, which reduces the dominance of the highest-count peaks but also weakens fidelity pressure in those regions relative to the original S2F objective.

We next asked whether part of this fidelity loss reflected missing DNA information rather than loss balancing alone. To test this, we introduced a DNA-aware profile-shape editor (Sec. 2.4.4; Fig. 4c; Fig. S3b, available as supplementary data at *Bioinformatics* online). To limit direct copying of visible DNA into edited outputs, editor DNA visibility was coupled to critic supervision as described in Sec. 2.4.4. Under the critic used for DDM and mask coupling, DNA-aware editing retained a substantial reduction in the masked DNA-decodability gap relative to the original S2F predictions (Fig. 5a), while partially recovering profile-shape fidelity relative to the DNA-free stage, especially in stronger peaks (Fig. 5b).

Finally, we asked whether the reduction observed in the DNA-free stage was specific to the critic used for the DDM objective. For this purpose, we retrained multi-headed cgLM critics on GM12878 ATAC-seq versus original and DNA-free edited AlphaGenome and BPNet profiles (Fig. 5c). We quantified the masked DNA-decodability gap on validation-set peaks, as described in Sec. 3.4. We observed that the separation between the original S2F predictions and the DNA-free edited profiles persists. This supports that the reduced masked DNA-decodability is not solely explained by idiosyncrasies of a single frozen critic.

These results position CGPSE as a preliminary framework with limited current scope. The reduction in masked DNA-decodability gap remained partial, and the accompanying fidelity cost was most evident in stronger peaks. As DDM is defined through a frozen critic, CGPSE may still inherit critic-specific failure modes, even though fresh-critic retraining provides one robustness check. CGPSE is also a post hoc, multi-stage framework that requires a pretrained S2F model, critic training, and editor training. We therefore interpret it as a preliminary investigation of whether the cgLM probe used to diagnose the gap (Sec. 3.2; Sec. 3.4) can also be repurposed to reduce it.

## 4 Discussion

We used track-conditional genome language models as a DNA-aware probe of DNA-track dependency in functional genomic tracks. Under this probe, experimental ATAC-seq and TF ChIP-seq showed distinct assay-specific behavior. In contrast, many S2F-predicted tracks exhibited a pronounced masked DNA-decodability gap relative to matched experimental tracks. This gap was observed across cell lines and across S2F model families, including both single-task and multi-task predictors.

Our control analyses further clarified how this gap behaves across evaluation regimes. In experimental ATAC-seq, peak regions were more decodable than nonpeak regions. Experimental TF ChIP-seq and control ChIP-seq remained weak overall, especially in the track-only regime. The DNA-only baseline also dropped markedly on dinucleotide-shuffled loci, confirming that shuffling disrupted recoverable sequence structure. In contrast, many cgLMs trained on S2F-predicted tracks remained highly decodable across peaks, nonpeaks, and dinucleotide-shuffled loci. They also showed much weaker regime dependence than the matched experimental tracks. The gap was not captured by standard DNA-agnostic profile-shape fidelity metrics such as JSD alone. Together, these findings indicate that DNA-aware probing reveals a property of S2F-predicted tracks that is complementary to conventional count and profile-shape fidelity.

At the same time, the masked DNA-decodability gap should be interpreted conservatively. Within the present study, it denotes a discrepancy revealed by the cgLM probe. Specifically, many S2F-predicted tracks supported easier masked nucleotide recovery than their matched experimental tracks under controlled DNA masking. The ChromBPNet comparison is particularly informative. ChromBPNet Tn5-denoised predictions behaved substantially closer to the experimental regime than several other S2F predictors. In contrast, cgLMs trained on the nonpeak-trained Tn5 bias component remained highly decodable on peaks. This suggests that staged, bias-factorized training can reduce the masked DNA-decodability gap. More broadly, our results suggest that DNA-aware evaluation provides a useful additional axis for comparing S2F models and training strategies.

Finally, we showed that this diagnostic can be turned into a critic-derived objective through DNA-dependency matching (DDM). This yielded the preliminary post hoc framework CGPSE, which partially reduced the masked DNA-decodability gap for AlphaGenome and BPNet predictions in GM12878 ATAC-seq, but with a profile-shape fidelity tradeoff.

## Supplementary Material

btag266_Supplementary_Data

## Data Availability

Experimental functional genomics data used in this study are publicly available from ENCODE under the following accessions: ENCSR868FGK (K562 ATAC-seq); ENCSR000EGM (K562 CTCF ChIP-seq); ENCSR000EHI (K562 control ChIP-seq); ENCSR637XSC (GM12878 ATAC-seq); ENCSR841NDX (GM12878 ELF1 ChIP-seq); and ENCSR956WYO (GM12878 control ChIP-seq). Publicly available ChromBPNet models used in this study are available from ENCODE under accessions ENCSR467RSV for K562 and ENCSR389HIH for GM12878. The ChromBPNet training and evaluation region sets used in this study are also available from ENCODE under accessions ENCFF991RUK (K562) and ENCFF971WEQ (GM12878). In-house trained checkpoints for the single-headed cgLM, multi-headed cgLM, and CGPSE components, together with preprocessed datasets generated in this study, are available at https://doi.org/10.5281/zenodo.19210955.

## Supplementary information

Supplementary figures and tables are available at *Bioinformatics* online.
