# Supplementary material for: DNA-aware evaluation and debiasing of sequence-to-function models
Source: Bioinformatics. 2026 Jul 7;42(Suppl 1):btag266. doi: 10.1093/bioinformatics/btag266 (PMC13340215; doi:10.1093/bioinformatics/btag266)
Supplement: btag266_Supplementary_Data [file btag266_supplementary_data.pdf]

Supplementary Material for DNA-aware evaluation and debiasing of sequence-to-function models

Doruk Cakmakci[1,2] and Yue Li[1,2,*]

[1] School of Computer Science, McGill University, Montreal, QC, Canada
[2] Mila – Quebec AI Institute, Montréal, QC, Canada
[*]Corresponding author. Yue Li, yueli@cs.mcgill.ca

S1 Supplementary Text

S1.1 Implementation details for CGPSE

This section provides implementation details for the Critic-Guided Profile Shape Editing (CGPSE) framework introduced in Sec. 2.4. It focuses on architecture, masking, optimization, and model-selection details that are omitted from the main text. The stage-specific training setup is illustrated in Fig. S3.

Critic input and DDM supervision masks During CGPSE, the critic always received a fully visible conditioning track and a partially visible DNA sequence. The critic DNA input mask M_i determined which nucleotide positions were hidden from critic input, and the critic supervision mask M_o determined which positions contributed to DNA-dependency matching.

Training used three critic mask configurations: (i) *track-only*, where all DNA positions were masked and all positions were supervised; (ii) *tied*, where critic input and supervision masks were identical; and (iii) *nested*, where the supervision mask was sampled first and extended to the critic input mask with additional masked DNA positions.

In the nested setting, the supervision-mask rate was sampled uniformly as $r_o \sim \mathcal{U}(0.10, 0.60)$. The critic-input mask rate was calculated as $\text{Beta}(2, 3) \times (0.80 - r_o)$, which ensured $M_o \subseteq M_i$. Training mixed the track-only, nested, and tied settings with probabilities $(0.1, 0.6, 0.3)$, respectively. Validation used two fixed tied-mask bins: 10%–20% masked DNA and 100% masked DNA (track-only).

DNA-free profile-shape editor

Architecture The DNA-free profile-shape editor was implemented as a debiasing autoencoder (DAE) operating only on log profile-shapes. It used one encoder for experimental inputs and one encoder for S2F-predicted inputs. Both encoders used the same architecture: a stem convolution of kernel size 23 followed by three strided downsampling blocks with kernel size 5 and channel progression $16 \to 32 \to 64 \to 64$, reducing the 1-Kbp input to length 125. Global mixing of length-reduced representations was performed by a single transformer block with 4 attention heads, dropout 0.1, feedforward multiplier 2.0, RoPE fraction 0.5, and RoPE theta 20000. The latent channel dimension was then reduced from 64 to 32, yielding a latent representation K_{free} of shape $B \times 32 \times 125$. The decoder mirrored the encoder with three upsampling blocks and a final projection to a single output track. Figure S3a summarizes the stage structure.

Training passes and loss routing The DNA-free DAE was trained with two passes. In the experimental-input mode, the model reconstructed the input experimental profile shape. In the S2F-input mode, it mapped the S2F-predicted profile shape toward the matching experimental profile shape. Let K_{exp} and K_{free} denote the bottleneck representations produced in the two modes. In addition to profile-shape supervision, these latent representations were aligned to encourage a shared latent space between experimental and S2F profile-shapes. In implementation, the latent-alignment term used a Huber loss with threshold 1.0 on bottleneck codes. DDM was applied to outputs from both modes under the frozen critic. Because both encoders were trainable, the latent-alignment term induced a bidirectional attraction between the experimental and S2F bottleneck representations. Although the DAE did not observe DNA directly, its outputs remained indirectly constrained by DNA–track dependency through critic supervision.

Optimization and model selection The DNA-free DAE was trained with AdamW using batch size 512, learning rate 10^{-3}, weight decay 10^{-3}, gradient clipping 1.0, and full-precision training. Training ran for up to 200 epochs without early stopping. Validation was performed at every epoch and used 10%–20% and 100% DNA-mask bins for critic-based metrics. Model selection used the total validation loss in the 100% DNA-mask bin. The selected DNA-free checkpoint was then frozen and reused in the DNA-aware stage.

DNA-aware profile-shape editor

Architecture The DNA-aware profile-shape editor was built on top of the trained DNA-free DAE. All DAE parameters were frozen and used as the base editor. The only trainable components were a profile-shape re-encoder, a masked-DNA encoder, and a latent editor operating on the frozen DAE bottleneck representation. The profile-shape re-encoder consumed the DNA-free edited log profile shape and used the same downsampling pattern as the DNA-free encoder, with channel progression $32 \to 64 \to 64 \to 64$. The masked-DNA encoder consumed a fused value-plus-mask tensor of shape $B \times 2 \times 1000 \times 4$ using a mask-aware convolutional stem with kernel size 23, followed by three downsampling blocks with kernel size 5. The resulting profile-shape and DNA features were added and passed through a bottleneck encoder to produce a context representation of shape $B \times 64 \times 125$. The latent editor took as input the frozen DNA-free bottleneck representation $K_{\text{free}} \in \mathbb{R}^{32\times125}$ and the context representation. After channel-wise concatenation and two SwiGLU projection blocks, it predicted FiLM-style modulation parameters (a, b) of the same shape as K_{free}, and the updated bottleneck representation was

$$K_{\text{aware}} = (1 + a) \odot K_{\text{free}} + b.$$

The updated bottleneck representation was decoded by the frozen DNA-free decoder to obtain the DNA-aware profile-shape output. The final latent-editor readout was initialized to zero so that the initial DNA-aware output matched the frozen DNA-free output.

Editor DNA input Masked DNA was represented as a fused value-plus-mask tensor. The first channel contained one-hot DNA values with masked positions set to zero. The second channel contained the DNA mask itself, where 1 indicated masked and 0 indicated visible positions.

Training and loss routing During DNA-aware training, the experimental route was used only as a fixed latent anchor. The trainable branch operated on S2F-derived profile-shapes only. Concretely, the frozen DAE first produced a DNA-free edited output and its bottleneck representation from the S2F-input mode. The profile-shape re-encoder and masked-DNA encoder then produced a context representation, the latent editor updated the frozen bottleneck representation, and the frozen decoder produced the DNA-aware profile-shape output. Gradients flowed only through the profile-shape re-encoder, masked-DNA encoder, and latent editor. Neither the frozen DAE nor the frozen critic received gradient updates. The DNA-aware stage used the same optimizer, training duration, validation bins, and count-scaling scheme as the DNA-free stage. Model selection again used the total validation loss in the 100% DNA-mask bin.

Gradient-ratio controller Both editing stages used the shared objective from Sec. 2.4.5. Count scaling used

$$w(N) = \sqrt{\max(N, c_{\min})}$$

with lower bound $c_{\min} = 133$, corresponding to the 75th percentile of total counts across training nonpeak loci. The same count-scaling rule was applied to the profile-shape cross-entropy term, the latent-alignment term, and the DDM term.

The coefficient λ_t was adapted online with a gradient-ratio controller to balance count-scaled DDM against the count-scaled profile-shape losses. Let g_{CE} and g_{DDM} denote exponential moving averages of the corresponding gradient norms. The controller updated

$$\lambda_t \leftarrow \text{clip}\left(\frac{g_{\text{CE}}}{g_{\text{DDM}} + \varepsilon}, \lambda_{\min}, \lambda_{\max}\right),$$

with initialization $\lambda_t = 1.0$, EMA smoothing coefficient 0.9, and clipping bounds $[\lambda_{\min}, \lambda_{\max}] = [0.1, 2000.0]$. Gradient norms were computed on the edited outputs and normalized by the square root of the number of elements.

In the DNA-free stage, profile-shape and DDM losses were applied to both experimental and S2F-input modes, while the latent-alignment term aligned their latent representations. In the DNA-aware stage, all three losses were applied only to the edited S2F branch, with the experimental route supplying the fixed latent anchor.

S1.2 Control experiments for the single-headed cgLM training setup

To further characterize the masked DNA-decodability gap between experimental and S2F-predicted tracks (Sec. 3.2), we performed three targeted control experiments with the single-headed cgLM setup (Fig. S6). We focused on GM12878 ATAC-seq and two DNA visibility regimes: light DNA masking (10–20% DNA mask) and the track-only regime (100% DNA mask). For each experimental ATAC-seq, ChromBPNet Tn5-denoised, AlphaGenome, and BPNet conditioning tracks, we retrained cgLMs which ablate the following design choices: (a) total-count scaling, (b) conditioning-track resolution, and (c) cgLM input window length. We summarize performance with cross-entropy at masked positions, which reflects both masked nucleotide prediction correctness and confidence.

To test whether total-count differences contribute to the masked DNA-decodability gap, we rescaled each S2F-predicted 1-Kbp profile to match the total count of the corresponding experimental track. Under both DNA visibility regimes, this count equalization had negligible effect on cgLM performance (Fig. S6a). This indicates that total-count differences are not a primary driver of the gap. We therefore used S2F predictions total count matched to respective experimental tracks in the remaining control experiments.

We next asked whether the masked DNA-decodability gap is concentrated at fine scales of the conditioning track. To do so, we constructed lower-resolution conditioning tracks by partitioning each 1-Kbp rescaled profile into non-overlapping bins of width 2, 4, or 8-bp, mean-pooling within each bin, and repeating the pooled values back to base resolution. To reduce sensitivity to bin boundaries, cgLMs were trained with shift augmentation. Under light DNA masking, coarsening the conditioning track to 8-bp narrows but does not eliminate the gap (Fig. S6b). BPNet- and AlphaGenome-conditioned cgLMs become less decodable, but remain more decodable than experimental and ChromBPNet Tn5-denoised settings. Under the track-only regime, the different conditioning tracks become more similar at 8-bp. These results indicate that the gap is strongest at fine scales, but is not limited to single-base resolution.

Finally, we tested the dependency of the masked DNA-decodability gap on local versus longer-range context by varying cgLM input window length from 16 to 512-bp. Under light DNA masking, the gap is already visible at 16-bp: BPNet- and AlphaGenome-conditioned cgLMs are substantially more decodable than cgLMs trained on experimental and ChromBPNet Tn5-denoised tracks (Fig. S6c). As window length increases, BPNet and AlphaGenome improve rapidly and approach near-deterministic decoding, whereas experimental and Tn5-denoised settings improve only modestly. In the track-only regime, increasing window length yields smaller gains and does not produce the same collapse toward near-zero cross-entropy. These results indicate that the gap is detectable even in short windows, but becomes much more pronounced when additional context is available and partial DNA is visible.

Together, these control experiments confirm that the masked DNA-decodability gap is not driven by total-count differences, but instead reflects structured differences in predicted profile shape under the cgLM probe. The resolution ablations indicate that the effect is strongest at fine scales, while remaining detectable after coarsening. The window-length ablations show that the effect is already present in short local context and is strongly amplified by longer-range context under partial DNA visibility. Collectively, these findings further constrain how the masked DNA-decodability gap should be interpreted (Fig. S6).

Supplementary Figures

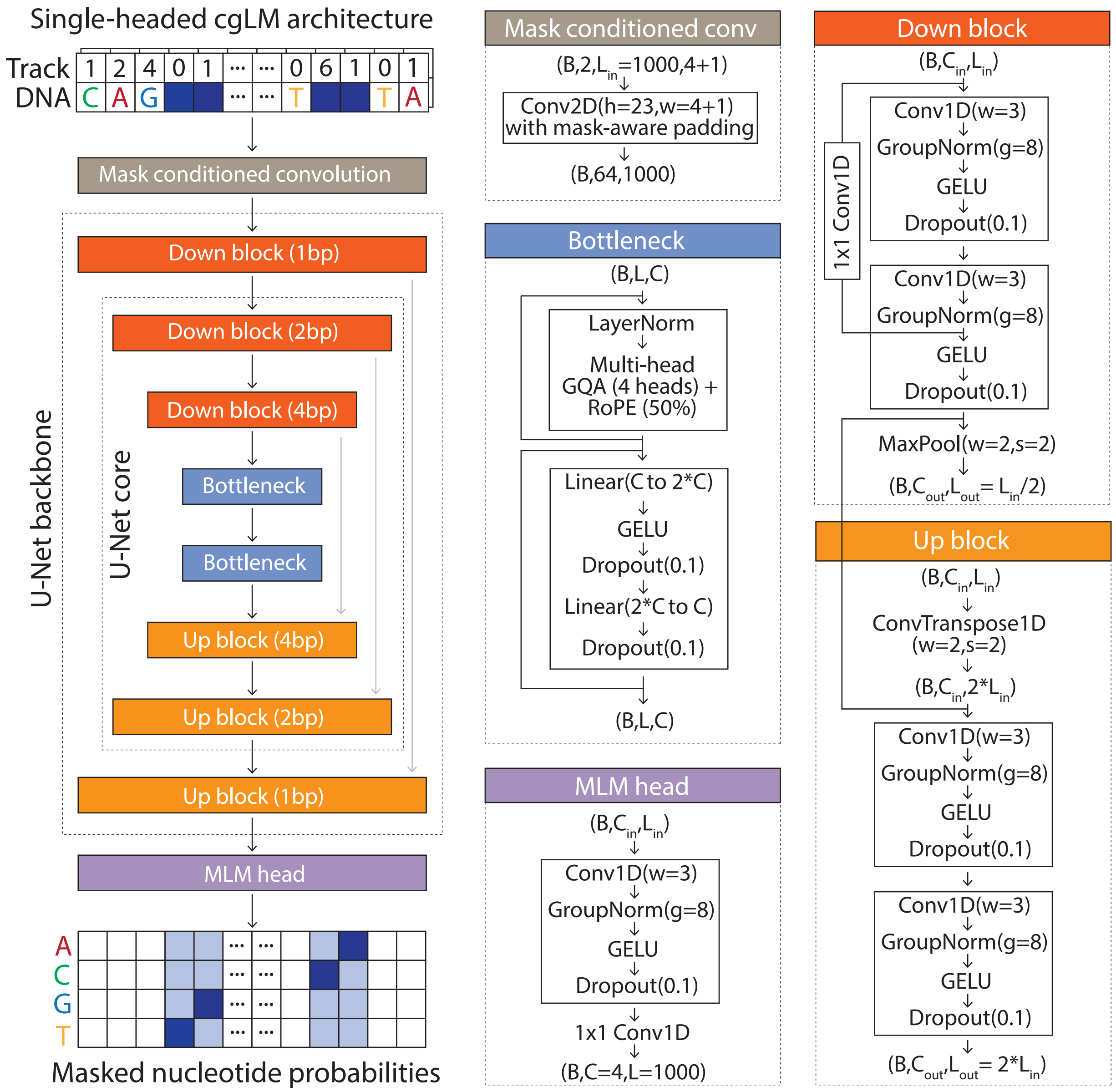

Fig. S1. Single-headed track-conditional genome masked language model (cgLM) architecture. Concatenated masked 1-Kbp DNA and track matrix first passes through a mask conditioned convolution of kernel size (23,4+1). Then a U-Net backbone with the three down blocks, two bottleneck blocks and three up blocks extracts per nucleotide embeddings. A lightweight MLM head predicts nucleotide probabilities. Bottleneck employs pre-norm transformer blocks.

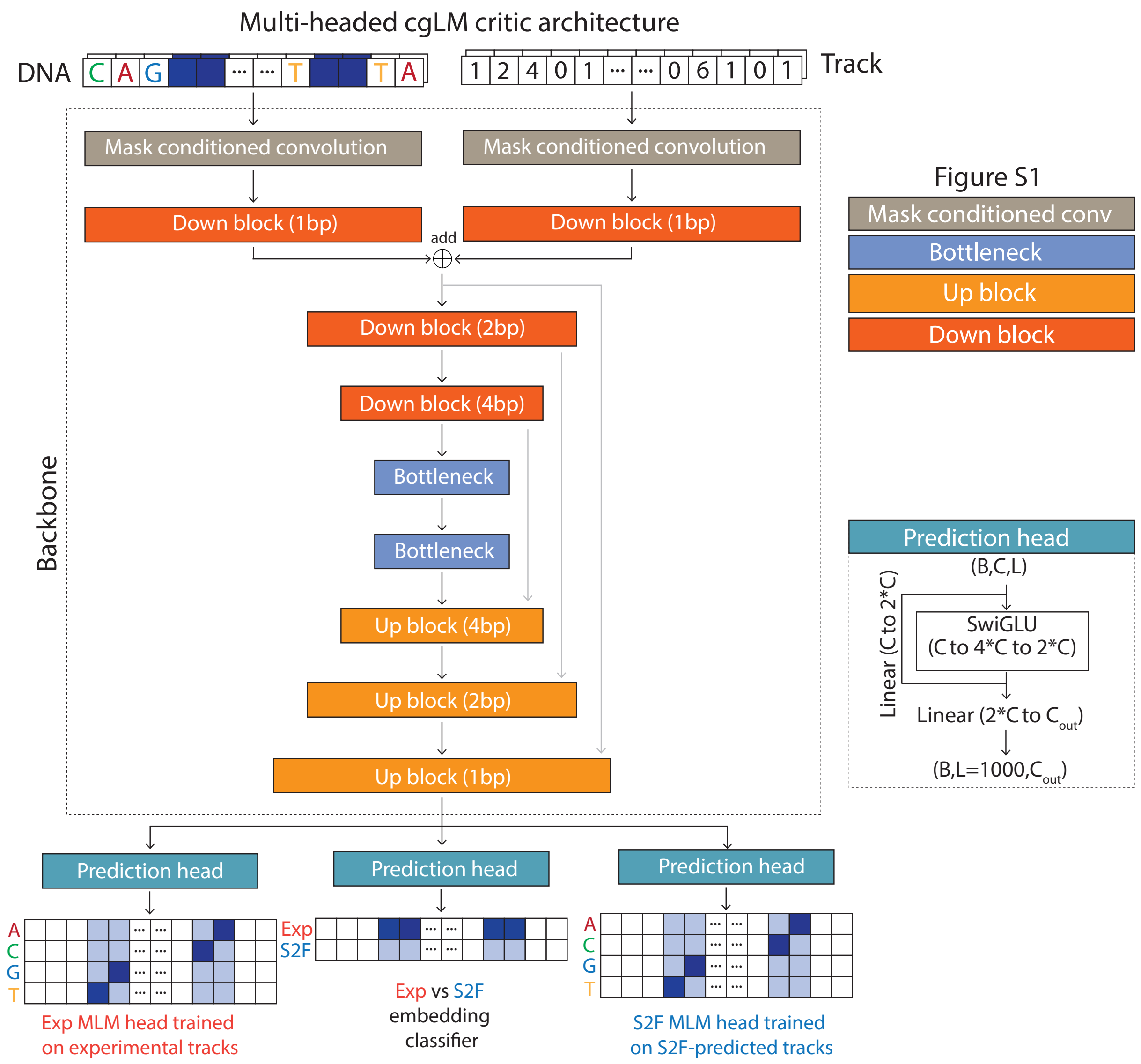

Fig. S2. Multi-headed track-conditional genome masked language model (cgLM) architecture. Masked 1-Kbp DNA and tracks are processed respective stems. The stems employ a mask conditioned convolution of kernel size (23,4+1), followed by a down block. Track and DNA stem outputs are added and passed to a U-Net backbone with the two down blocks, two bottleneck blocks and three up blocks. Backbone outputs are per nucleotide embeddings. Three prediction heads each embedding to outputs.

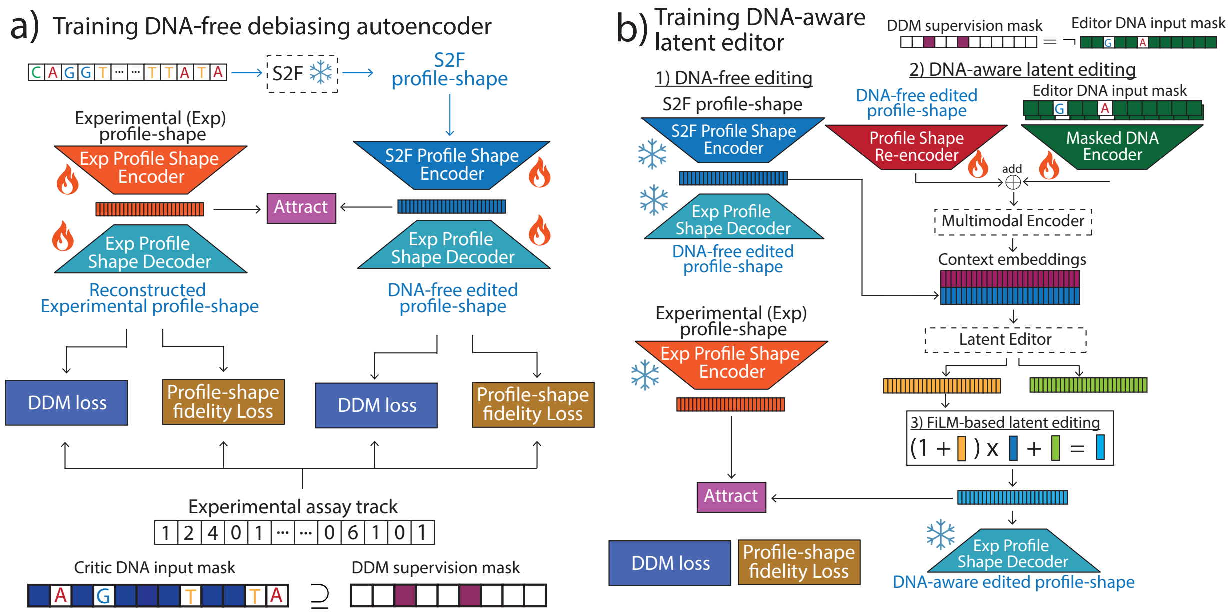

Fig. S3. Stage-specific training setup for Critic-Guided Profile Shape Editing (CGPSE). (a) DNA-free debiasing autoencoder (DAE). The DNA-free editor operates only on log profile-shapes and is trained in two modes. In the experimental-input mode, the experimental profile-shape is encoded and reconstructed. In the S2F-input mode, the S2F-predicted profile-shape is encoded and mapped toward the matched experimental profile-shape. The two modes use separate encoders, a shared decoder, and a latent-alignment loss that pulls their bottleneck representations toward a shared latent space. Profile-shape fidelity and DNA-dependency matching (DDM) losses are applied to outputs from both modes under the frozen critic. Although the DAE does not observe DNA directly, its outputs remain indirectly constrained by DNA–track dependency through critic supervision. During training, the critic receives a fully visible conditioning track and partially visible DNA under track-only, tied, and nested masking configurations. (b) DNA-aware latent editor. The trained DNA-free DAE is frozen and used as the base editor. The frozen DNA-free edited profile-shape and bottleneck representation from the S2F-input mode are combined with masked DNA through a profile-shape re-encoder, a masked-DNA encoder, and a latent editor. The re-encoder and masked-DNA encoder produce a context representation, and the latent editor predicts FiLM-style modulation parameters that update the frozen DNA-free bottleneck before decoding, yielding a DNA-aware edited profile-shape. In this stage, the experimental route provides only a fixed latent anchor, and all trainable updates are restricted to the S2F-derived branch. Gradients flow only through the profile-shape re-encoder, masked-DNA encoder, and latent editor, while the frozen DAE and frozen critic are not updated. To limit direct copying of visible DNA into edited outputs, editor DNA visibility is coupled to critic supervision through $M_{\mathrm{edit}} = \neg M_o$.

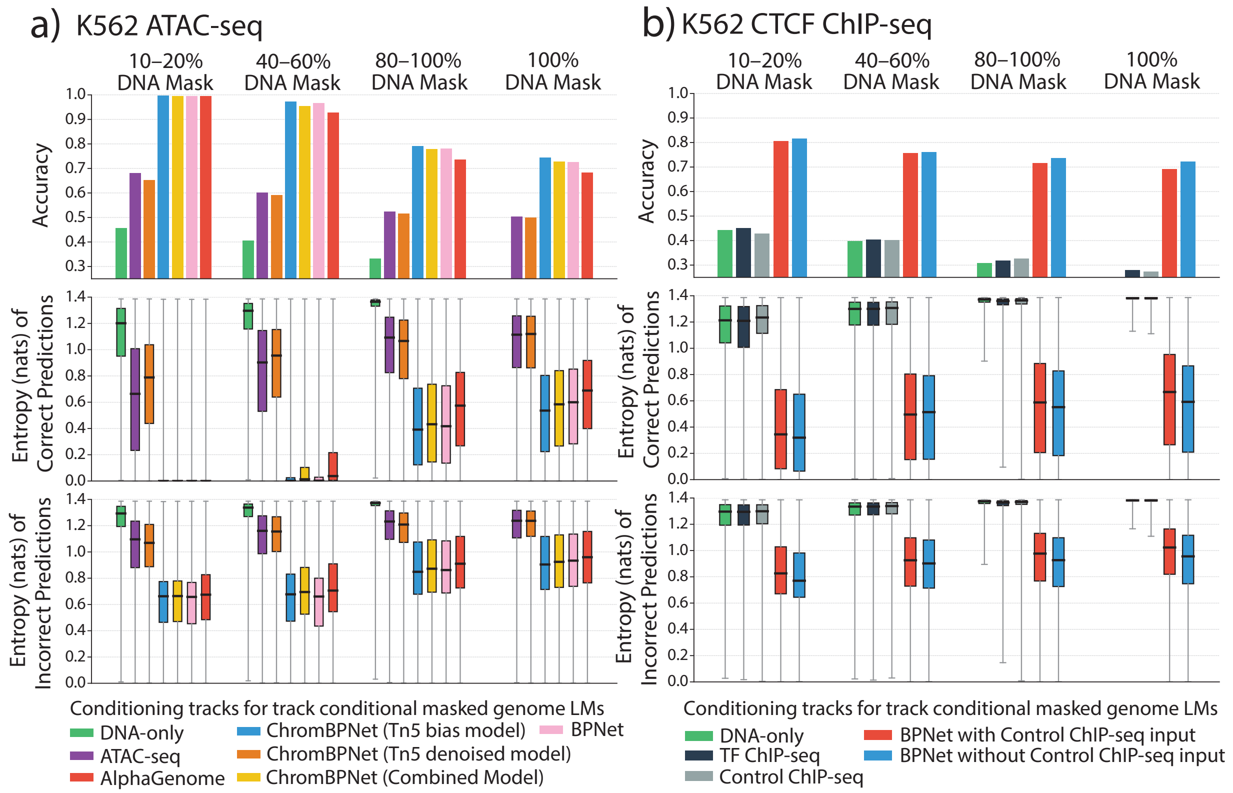

Fig. S4. (a) Test set (peaks) performance of cgLM and DNA-only baseline (green) trained on K562 ATAC-seq (purple) as well as S2F-predicted tracks (others) for different DNA masking regimes. Each bar corresponds to a different model. (b) Test set (peaks) performance of cgLM and DNA-only baseline trained on K562 CTCF ChIP-seq (black), control track (grey) and predictions from BPNet with (red) and without (blue) control input.

Group	Conditioning tracks for Track-Conditional Masked Genome LMs	Masked nucleotide prediction accuracy (%): 10-20% DNA mask				100% DNA mask				Median JSD between experimental and S2F profile shapes	
		Peaks	Nonpeaks	Shuffled peaks	Shuffled nonpeaks	Peaks	Nonpeaks	Shuffled peaks	Shuffled nonpeaks	Peaks	Nonpeaks
K562 ATAC-seq	DNA-only	45.8%	48.7%	33.3%	33.4%	NA	NA	NA	NA	NA	NA
	ATAC-seq	68.2%	53.9%	NA	NA	50.3%	39.3%	NA	NA	NA	NA
	ChromBPNet (Tn5 denoised model)	65.4%	66.3%	60.7%	60.4%	50.1%	51.6%	46.4%	46.3%	0.597	0.742
	ChromBPNet (Tn5 bias model)	99.8%	99.6%	99.8%	99.8%	74.4%	74.7%	70.7%	70.8%	0.395	0.600
	ChromBPNet (Combined model)	99.4%	99.4%	99.6%	99.6%	72.8%	73.7%	69.4%	69.6%	0.341	0.599
	BPNet	99.6%	99.0%	99.7%	99.7%	72.7%	73.3%	69.3%	69.4%	0.341	0.600
	AlphaGenome	99.6%	98.2%	97.6%	97.6%	68.3%	67.8%	NA	NA	0.331	0.593
K562 CTCF ChIP-seq	DNA-only	44.2%	48.3%	33.4%	34.1%	NA	NA	NA	NA	NA	NA
	TF ChIP-seq	45.1%	50.3%	NA	NA	28.0%	27.2%	NA	NA	NA	NA
	Control ChIP-seq	42.8%	46.3%	NA	NA	27.4%	26.4%	NA	NA	NA	NA
	BPNet without Control ChIP-seq input	82.0%	85.1%	82.5%	82.8%	72.5%	76.9%	72.8%	73.2%	0.661	0.823

Fig. S5. cgLMs recover K562 peak, nonpeak and shuffled DNA sequences from S2F-predicted tracks with comparable accuracy. Rows are grouped by assay (K562 ATAC-seq and CTCF ChIP-seq), and list the conditioning tracks used for track-conditional genome language models. Columns are grouped into masked nucleotide prediction accuracy at 10–20% DNA mask and at 100% DNA mask, each with separate subcolumns for peaks, nonpeaks, shuffled peaks, and shuffled nonpeaks. The final column group reports the S2F predicted profile-shape fidelity metric (median JSD) on peaks and nonpeaks. NA indicates settings not applicable (e.g., shuffled sequence evaluation with experimental assay tracks).

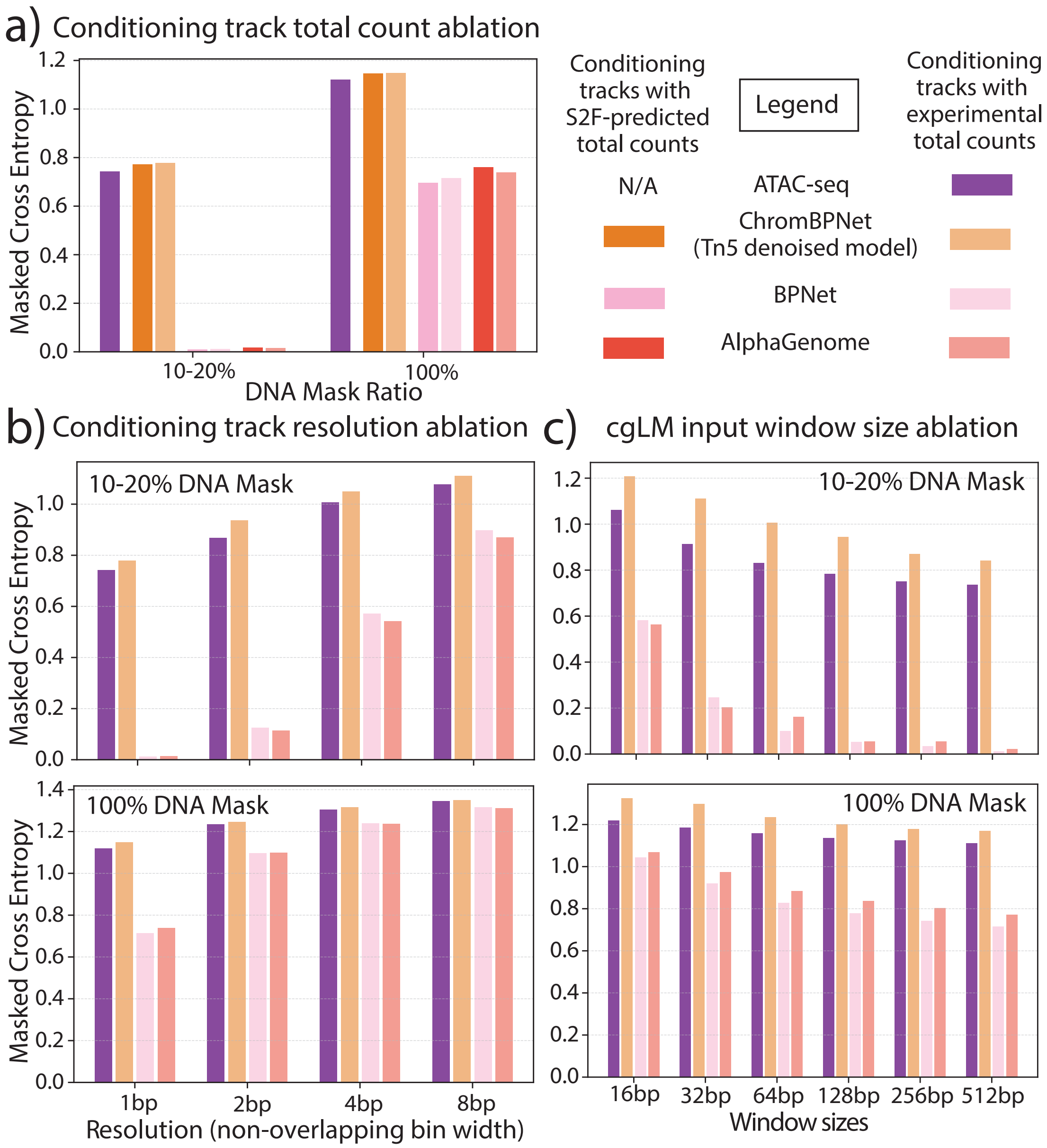

Fig. S6. Control experiments for models trained on GM12878 ATAC-seq (Sec 3.1 and 3.2). (a) Difference between total count predictions from S2F and experimental tracks does not contribute to masked DNA-decodability gap. (b) cgLM training on coarser resolutions suggest the sequence-linked structure is observable at multiple-scaled but concentrated at finer scales. (c) Screening a range of window lengths cgLMs indicate that the structure is auditable to a non-negligible extent in 16-bp windows but benefits from longer range context.

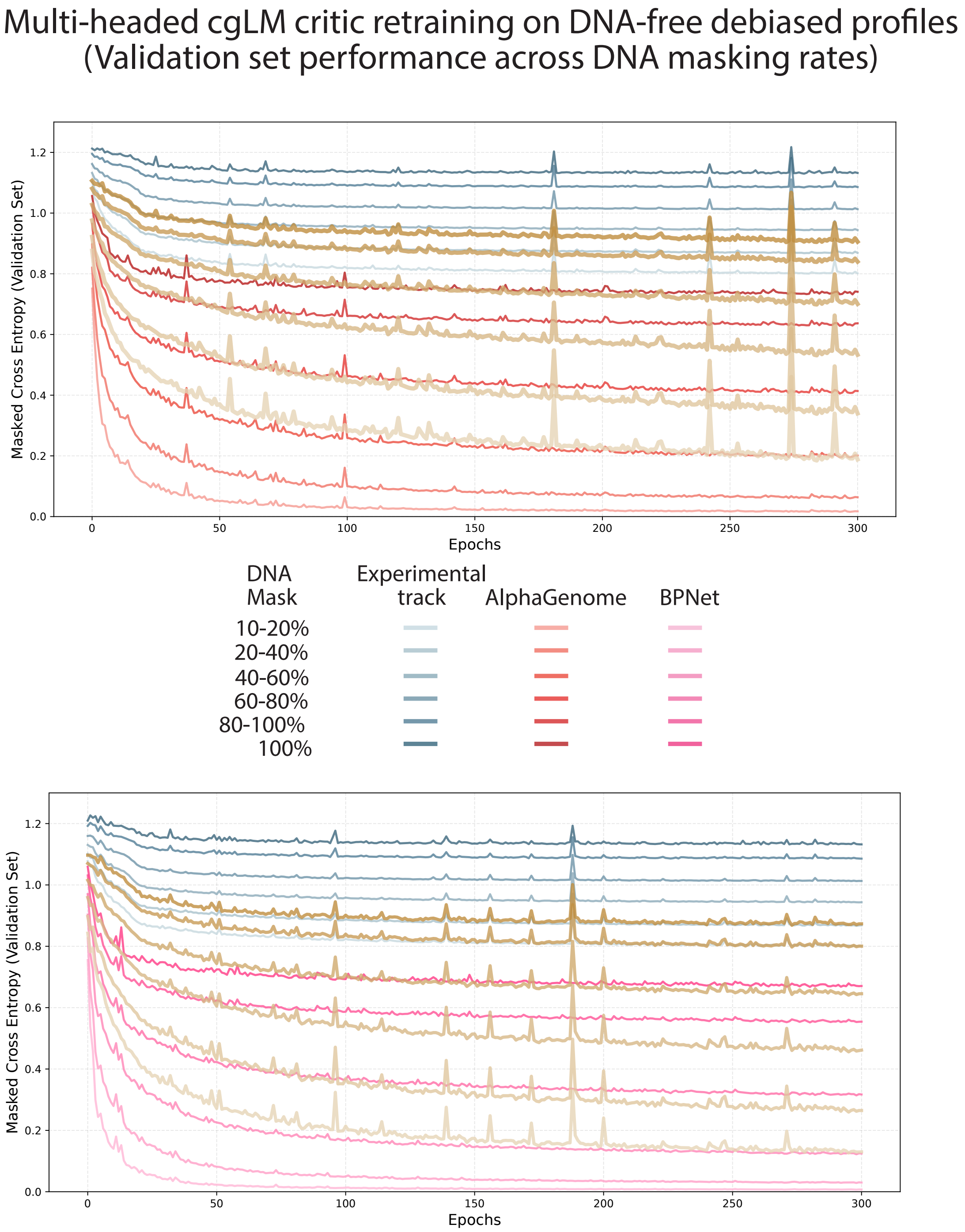

Fig. S7. Validation performance of the multi-headed cgLM critic retrained on DNA-free debiased profiles over 300 epochs. The top panel compares the experimental track to the debiased AlphaGenome predictions, and the bottom panel compares the experimental track to debiased BPNet predictions. Performance is shown across six DNA masking rate regimes ranging from 10-20% to 100%.
